# Emerging Paradigms in Bioengineering the Lungs

**DOI:** 10.3390/bioengineering9050195

**Published:** 2022-05-01

**Authors:** Raxshanaa Mohgan, Mayuren Candasamy, Jayashree Mayuren, Sachin Kumar Singh, Gaurav Gupta, Kamal Dua, Dinesh Kumar Chellappan

**Affiliations:** 1 School of Pharmacy, International Medical University, Kuala Lumpur 57000, Malaysia; raxshanaa.mohgan@student.imu.edu.my; 2 Department of Life Sciences, School of Pharmacy, International Medical University, Kuala Lumpur 57000, Malaysia; mayurencandasamy@imu.edu.my; 3 Department of Pharmaceutical Technology, School of Pharmacy, International Medical University, Kuala Lumpur 57000, Malaysia; jayashree@imu.edu.my; 4 School of Pharmaceutical Sciences, Lovely Professional University, Jalandhar-Delhi G.T Road, Phagwara 144411, India; sachin.16030@lpu.co.in; 5 Australian Research Centre in Complementary and Integrative Medicine, Faculty of Health, University of Technology Sydney, Sydney 2007, Australia; kamal.dua@uts.edu.au; 6 School of Pharmacy, Suresh Gyan Vihar University, Jaipur 302017, India; drgaurav.gupta@mygyanvihar.com; 7 Department of Pharmacology, Saveetha Dental College, Saveetha Institute of Medical and Technical Sciences, Saveetha University, Chennai 600077, India; 8 Uttaranchal Institute of Pharmaceutical Sciences, Uttaranchal University, Dehradun 248007, India; 9 Discipline of Pharmacy, Graduate School of Health, University of Technology Sydney, Sydney 2007, Australia

**Keywords:** lung bioengineering, scaffolds, lung-on-a-chip, lung transplantation, artificial lung, bioprinting

## Abstract

In end-stage lung diseases, the shortage of donor lungs for transplantation and long waiting lists are the main culprits in the significantly increasing number of patient deaths. New strategies to curb this issue are being developed with the help of recent advancements in bioengineering technology, with the generation of lung scaffolds as a steppingstone. There are various types of lung scaffolds, namely, acellular scaffolds that are developed via decellularization and recellularization techniques, artificial scaffolds that are synthesized using synthetic, biodegradable, and low immunogenic materials, and hybrid scaffolds which combine the advantageous properties of materials in the development of a desirable lung scaffold. There have also been advances in the design of bioreactors in terms of providing an optimal regenerative environment for the maturation of functional lung tissue over time. In this review, the emerging paradigms in the field of lung tissue bioengineering will be discussed.

## 1. Introduction

Each year, more than 4600 lung transplantations are carried out globally, of which 55% are performed in North America and 36% in Europe [1]. The replacement of diseased lungs with a pair of healthy ones in lung transplantation (LTx) increases both the life expectancy and quality of life in patients [2,3]. However, this approach is only preferred when patients are not responding to medications. The most common indications where transplantation is opted for are in the end stage of diseases such as chronic obstructive pulmonary disease (COPD), cystic fibrosis (CF), interstitial pulmonary fibrosis, and primary pulmonary arterial hypertension [3,4]. In terms of pulmonary malignancy, it was found that a select group of patients undergoing Ltx had desirable outcomes [5]. In patients with severe unresolving COVID-19 associated with acute respiratory distress syndrome, lung transplantation was the only option [6].

The assessment process for LTx is usually long and complicated. The American Thoracic Society and International Society for Heart and Lung Transplantation have developed clear patient selection criteria, which consider age and severity of the disease where both psychological and clinical aspects are taken into consideration. If the patients are not responding to medications or if no medications are available for the medical condition, physicians will start to consider Ltx as a viable treatment option [7]. The lung allocation score, which was introduced by the USA and adopted by some countries in Europe with slight modifications as per their needs, has been shown to diminish mortality rates in patients on the waiting list for transplants [7,8,9]. The introduction of lung allocation scores drastically reduced the waiting time for system allocation, and it is based on the intensity of the need and the chances of post-transplant survival [1].

However, Ltx is a complex therapy with a significant risk of perioperative morbidity and mortality and has the worst outcome of all solid organ transplantations. It offers patients 78% 1-year survival, 63% 3-year survival, and 51% 5-year survival [10]. Many challenges involving Ltx that have yet to be overcome include expansion of the lung donor pool, inducing tolerance, bridging patients to transplant, and preventing a multitude of complications, such as primary graft dysfunction, cellular and antibody-mediated rejection, chronic allograft rejection, and infections [11]. In the first-week post-transplantation, complications such as reperfusion injury and infection may occur, followed by, in the second week, the possibility of organ rejection and infection. Chronic rejection and infection are more likely and occur earlier in lung allografts. This might be due to causes such as direct exposure to the external environment, increasing the susceptibility of direct injury from inhaled foreign material and infection. Furthermore, since lung allografts lack lymphatic drainage and innervations with an abnormal mechanism of mucociliary clearance, ineffective foreign material or microorganisms’ removal is expected to be the cause of infection [10]. Hence, in terms of having an improved end-of-life treatment in patients requiring lung transplantation, currently, new efforts are being made in the field of bioengineering technology.

**Figure 1:**
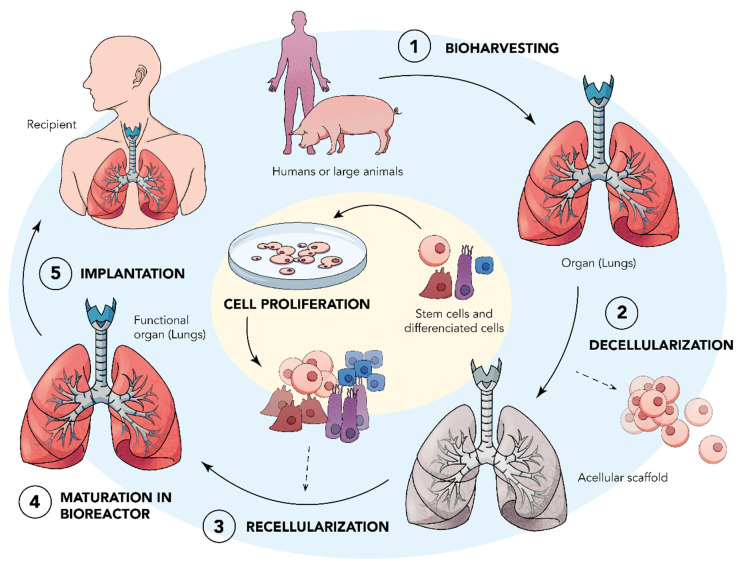
A schematic representation of developing bioengineered lungs.

In bioengineering, ex vivo strategies of transplantation that could address the issue of donor organ shortage via conventional Ltx are being investigated thoroughly. Pre-clinical studies are being conducted that utilize biologically derived or synthetic scaffolds that are seeded with autologous cells from the transplant recipient. In the generation of scaffolds, a multitude of different technologies has been developed, such as decellularization for biological scaffolds and advanced manufacturing processes such as casting, cryogelation, microfabrication, and electrospinning techniques for synthetic scaffolds (Figure 1) [12]. Upcoming advancements, such as xenotransplantation, are also being studied as an alternative approach to allogenic Ltx or as a bridge to allotransplantation by extending the time for a donor–recipient match to be found. There is ongoing research in genetic engineering to prevent acute thrombotic and severe inflammatory reactions occurrences via pig and primate xenotransplantation [13,14]. Additionally, bioprinting has emerged as a popular field of study for the engineering of biocompatible scaffolds for the purposes of transplantation. The ability to systematically deposit layers of biologically active cells and matrix material for engineered tissue through 3D rapid prototyping and inkjet valve-based printing systems could possibly create alveolar and microvascular structures of polymeric materials. It has also been reported that bioprinters may be able to produce cells and extracellular matrix (ECM) simultaneously to create a complete scaffold [14,15].

In this review, the introduction of various scaffolds together with their manufacturing processes, including decellularization and recellularization strategies, are discussed. Furthermore, recent bioreactor strategies that aid in successful lung bioengineering and the regulatory and ethical implications in this field of research are also discussed.

## 2. Lung Scaffolds

LTx is an option for the end stages of lung diseases because of the limited regeneration capacity of adult lung tissue [16]. The lack of suitable donors is a major issue, and it is being bridged by tissue engineering. Damaged tissues can be regenerated using cells from the body along with materials like scaffolds as templates to facilitate the development of newer tissues using the tissue engineering technique (Figure 1) [17]. The scaffolds offer a stencil for the redevelopment of defects. They are three-dimensional (3D), porous, fibrous, or permeable biomaterials intended to permit the transport of body liquids and gases and promote cell interaction and ECM deposition with minimum inflammation and toxicity while biodegrading at a certain controlled rate [18]. The characteristics necessary to be considered while choosing a scaffold to develop the lung tissue cannot be ignored. The physiological function of the lungs, for example, the exchange of gasses, should remain intact even with the use of scaffolds which is closely related to the properties such as strength, elasticity, cellular remodeling, geometry, cellular movement, nutrient transfer, and removal of waste, etc. These properties should be primary considerations when choosing a scaffold for the development of lung tissue [19].

In addition to these, a crucial component in maintaining lung scaffolds is the sterilization of decellularized lung matrix [20]. Sterilization is essential for eliminating all viable traces of microbes that may still be living on the scaffolds. Unlike natural living lungs that are protected from pathogens, these decellularized lung scaffolds are devoid of protective organelles such as macrophages and other immune-modulating substances. This may result in extremely dangerous end products in recipients who are already on immunosuppressants [21]. Thus, it is imperative that such lung scaffolds are sterilized with proper care [22]. Perfusion of the lung matrix in peracetic acid (PAA) is the common method adopted for sterilization [23]. PAA is reported to be effective in eradicating all forms of microorganisms and pathogens [24]. Recently, there has been much interest in the use of supercritical carbon dioxide (ScCO_2_) for the sterilization of lung scaffolds [25]. A number of reports suggest that ScCO_2_ is far superior to other known sterilizing agents as this technique acts deeply inside the matrix components and also works at lower temperatures. It is also reported to be non-toxic to the lung matrix components.

Another effective sterilization technique reported for lung scaffolds is photodynamic therapy (PDT). This technique requires the use of photosensitizer, which is essentially the key component in this technique [26]. Although this tool is gaining much interest, nevertheless, in-depth studies are required to prove the clinical utility of this technique. Apart from these, other common methods for the sterilization of lung scaffolds reported are the use of gases, namely, ethylene oxide or gamma rays, and electron particles [27]. The penetrative capability of ionizing radiation gives them an added advantage among the agents that are employed for the sterilization of lung scaffolds.

Apart from the general regulation of the extracellular volume inside the lung scaffolds, the osmolarity and pH of the matrix also need to be maintained at optimal levels. Large variations in either the pH or the osmolarity may cause the cells to behave in an extremely abnormal manner. This may also lead to the destruction of the lung matrix architecture, which may eventually affect the physiology of the scaffolds adversely.

One of the major challenges with whole lung transplantation is graft rejection, where the recipient system does not accept the transplanted lung. This is a slow, long-term process. Long-term rejection may lead to the development of a condition known as bronchiolitis obliterans syndrome (BOS), which is represented by the progressive obliteration of the small airways. Lung scaffolds are prone to be rejected by the immunological system after transplantation. Thus, it is important to consider the factor of immunogenicity. It is common to observe a strong immunological response in the recipient. Therefore, it is of utmost importance to manage the factors associated with graft rejection [28]. The most widely accepted strategy for minimizing rejection is to induce immunosuppression. In addition, certain biocompatible constructs and scaffold materials will be key to minimizing scaffold rejection. Recently, it has been discovered that certain stem cells are responsible for the primary cause of graft rejection in lung transplants. Major histocompatibility complex also plays a significant role in the rejection of the scaffold. Antibody-mediated rejection (AMR) is another cause of lung scaffold rejection. Inducing immunological tolerance is another strategy that has been reported by several authors to overcome chronic rejection [29].

### 2.1. Acellular Scaffolds

The use of human-derived acellular lung tissue scaffolds is a recent advance in lung bioengineering, allowing the study of lung tissue repair and reconstruction [30]. Scaffolds with a 3D structure facilitating cellular adhesion are considered an ideal and efficient template suitable to repopulate specific cells. Such properties are seen in acellular ECM scaffolds. They are lungs from which the cells have been destroyed and removed, thereby providing a scaffold from the skeleton of the lung. This structure supports the growth of adult, stem, or progenitor cells into functional lung tissues. Evidence has been reported that the survival of the engineered tissues after transplantation supports limited gas exchange, as well [18,19]. Acellular scaffolds developed from animals and patients are currently being used to explore human disease ex vivo as a new 3D model [31,32].

### 2.2. Artificial Scaffolds

Artificial scaffolds have been used as a supporting structure for the domination of cell growth in the repair of impaired tissues or organs such as the lung. Synthetic polymers such as poly-glycolic acid (PGA), poly lactic-co-glycolic acid (PLGA), poly-l-lactic acid (PLLA), polyvinyl alcohol (PVA), and polyethylene glycol (PEG) can be used to make artificial scaffolds. Importantly, to avoid adverse reactions, materials used to generate artificial scaffolds should be non-immunogenic, biocompatible, non-toxic, chemically stable, and well tolerated by the host after implantation [33].

## 3. Updates on Acellular Lung Scaffold Manufacturing

The foundational function of the lung and its physical properties are dependent on the ECM composition, which influences strength, flexibility, and elasticity. The interstitial lung parenchyma is mainly composed of collagen I, III, and elastin. The primary function of these components is to form the mechanical scaffold, which aids in maintaining the lung structure during ventilation. Cell removal from the lung might change the composition of ECM, thereby affecting the physical characteristics of the scaffold material [19]. However, the derivation of cell-free ECM was conducted decades ago by Lwebuga-Mukasa and colleagues, who first described the generation of acellular lung scaffolds in 1986 using an in vivo model, and this field has been growing rapidly in the recent years [20]. Hence, it is important to ensure that decellularization techniques performed are good enough to retain key ECM components while facilitating the removal of nucleic acids and cell debris [19]. Repopulation of the decellularized scaffold must also be optimized in order to produce a functional lung. Therefore, improved culture techniques are required to enhance complete recellularization of the lung surface area with viable, functioning, and differentiated cells [34].

There are several factors that play a major role in the selection of an ideal polymer for the manufacture of lung scaffolds. The use of synthetic and natural biopolymers has been favored by several authors. One of the key factors is the porosity of the polymer used. Porosity decides the extent of cell growth, vascularization, and attachment within the matrix [35]. Thus, an optimally porous material is crucial in the manufacture of the scaffolds. Another important factor of an ideal polymer material is the ability to functionalize. This may decide how strong the matrix is constructed. Orientation of the polymer fibers is another factor that needs to be considered. Reduction in the amount of degradation is also another factor that has been reported by several authors.

### 3.1. Decellularization

The primary focus of any decellularization technique is to remove the endogenous cell population while retaining the macro architecture along with the ECM composition of the lung. Effective decellularization techniques are dictated by factors, including tissue organization, density, or organ structure. In terms of decellularization of the lung, the aforementioned factors are crucial to note since the tissue density and structure vary considerably among main stem bronchi, bronchioles, and distal lung [36]. Various techniques have been used to decellularize lung tissue, such as using chemical, physical agents, and enzymes.

The most common chemical decellularization technique is using surfactants, which work by lysing cells via disarranging the phospholipid membrane [37]. Surfactants can be classified according to their charge, such as ionic, non-ionic, or zwitterionic. Sodium dodecyl sulfate (SDS) is a widely used ionic surfactant that efficiently removes cells and genetic materials. Studies have reported that treatment with SDS has met the standard requirements of complete cell removal and elimination of at least 90% of host DNA from rat, porcine, and human lungs [38,39]. However, the decellularized ECM of porcine and human lungs was more fibrous compared to the structure of smooth native tissue [38]. Glycosaminoglycans (GAGs) and growth factors such as vascular endothelial growth factor (VEGF) were reduced due to treatment with SDS, thereby affecting the biochemical cues regulating cellular function. Structural protein and components damage not only prevents the cells from inhabiting the tissue as before but will also inhibit the full retention of its mechanical properties [40]. SDS is more difficult to wash off due to its ionic nature; hence, often, the non-ionic surfactant Triton X-100 is used to remove the remnant of SDS [39]. Moreover, Triton X-100 can also be used as a decellularizing agent alone, and since it is non-ionic, it is less harsh than SDS, ultimately being less damaging to the structural tissue integrity. Together with ammonium hydroxide, it could completely remove all DNA while maintaining a high amount of collagen I compared to SDS [41]. Moreover, sodium deoxycholate (SD) is another ionic surfactant that functions by solubilizing the cell membrane, but unlike SDS, SD produces highly biocompatible scaffolds. Complete cell removal using SD was observed in a perfusion treatment of rat lungs, and at the same time, a greater amount of myosin was retained when using SD compared to other surfactant treatments [39]. Other than that, zwitterionic detergents such as 3-[(3-cholamidopropyl) dimethylammonio]-1-propanesulfonate (CHAPS) have also been used in decellularizing the lung tissue, whereby human and porcine-derived lung tissues and rat lungs, when treated with 8 Mm CHAPS solutions, exhibited complete decellularization [38,39]. However, it was reported that the cytoplasmic proteins had remained, but not all cellular debris was removed. Due to the non-denaturing properties of CHAPS, ECM proteins such as collagen and elastin were preserved, allowing the lung tissue to retain its compliance [39,42].

In general, most protocols last from 1 to 7 days. Many protocols of decellularization incorporate additional washes and incubations to remove organic components, which are difficult to remove using other detergents. The most widely used technique is using hypertonic solution (1 M NaCl) for cell lysis, or DNase/RNase to clear residual DNA and RNA. To date, there is no agreement on the best route of administration and removal of decellularizing agents since both vascular-only perfusion and a combination of vascular and airway perfusion have produced acellular scaffolds that are capable of supporting recellularization [12].

### 3.2. Recellularization

The lung is a complex organ composed of more than 40 different cell types that perform a variety of functions, including gaseous exchange, ciliary clearance of inhaled foreign objects, and immune system surveillance [43]. This complexity makes the generation of bioengineered lungs a complicated process whereby recellularization of the decellularized ECM must be optimized to produce a functional tissue or organ [41,44]. Recellularization is defined as the repopulation of acellular ECM scaffolds with specific cell types that are functional and transplantable based on the specific function of the organ [45]. The main cell sources that are currently being investigated include primary tissue-isolated progenitor cells, differentiated pluripotent stem cells, and mesenchymal stem cells (MSCs) [46,47,48,49].

Organ-specific on-site endothelial cells (EC) might represent the most promising cell source for microvascular lung generation as EC derived from lungs possess significant genome expression levels and transcriptomes [50]. Accordingly, lung microvascular ECs (LMVECs) have been used for recellularization in rat models; for example, Niklason’s group has seeded rat LMVECs with epithelial cells (EpCs) onto decellularized lung. When LMVECs were injected into the pulmonary artery of acellular scaffolds, they adhered throughout the scaffold [51,52]. Furthermore, cell lines, including human umbilical vein endothelial cells (HUVECs) and A549 alveolar EpCs, have been used in numerous experiments to demonstrate biocompatibility with the decellularized scaffold. These cells were shown to possess less translational potential as they are not patient-specific and may not be derived from normal tissues [53]. A study has also reported that HUVECs had lower levels of proliferation and a higher level of apoptosis compared to LMVECs [54].

Moreover, the use of induced pluripotent stem cell (iPSC)-derived cell populations is being broadly investigated as a promising cell source for ECs, thereby having the potential to overcome the need for primary lung tissue [30,55]. Human iPSC-derived ECs were applied to rat and human lung scaffolds resulting in the establishment of perfusable vascular lumens. In addition, although maintaining the genome integrity of the iPSCs and the differentiated cells is still an uncertainty, it may be possible to correct donor-specific genetic diseases such as CF in the iPSC state prior to differentiation [56]. A recent study has also demonstrated that human iPSCs can be differentiated into cells expressing a distal pulmonary epithelial cell immunophenotype and seeded into acellular human lung scaffolds [57]. Even though the clinical use of iPSCs is an exciting approach, it remains under development, and there are concerns regarding the tumorigenic potential of pluripotent cells [12].

Lastly, MSCs’ potential in repopulating various acellular scaffolds is being widely studied alongside the aforementioned cell types. MSCs are multipotent stromal cells that possess a self-renewal and differentiation capacity in which their binding with scaffolds occurs by the interaction of cellular integrins with different ECM proteins [58]. Mouse bone marrow-derived MSCs readily adhered to residual ECM after intratracheal inoculation into decellularized whole mouse lungs. It was found that MSCs spread more diffusely throughout the lung and appeared to grow in regions enriched in type I collagen, type IV collagen, and laminin but not in fibronectin [59]. Moreover, studies have indicated that MSCs derived from human bone marrow and human adipose tissue were attached to and proliferated within lung tissue scaffold. It also demonstrated the ability to differentiate toward lung epithelial phenotypes, thereby presenting MSCs as an important cell type for lung regeneration [60].

## 4. Artificial Lung Scaffolds

Due to recent advances, synthetic materials are an attractive candidate to use as scaffolds in the development of tissue engineering. Myriad synthetic polymers such as PLLA, PGA, and PLGA have been used in an attempt to produce artificial lung scaffolds. Although synthetic polymers can be fabricated successfully with a tailored architecture, there are drawbacks, such as the risk of rejection due to reduced bioactivity [16]. It has also been reported that the degradation process of PLLA and PGA by hydrolysis produces carbon dioxide, causing a reduction in the local pH, which can result in cell and tissue necrosis [61]. However, PGA efficiently functioned as a patch grafted to the incised lung of the rat model. The adipose-derived stem cells seeded PGA succeeded in regenerating vascular and alveolar tissues [62]. PDLLA is another biodegradable hydrophobic polymer of lactic acid that is favored to be used as a lung scaffold due to its elastic property [16]. Therefore, it is important to choose artificial materials which have biochemical and biomechanical properties and micro and nanoscale structures that mimic the native ECM of lungs [63]. Comparisons between acellular and artificial scaffolds can be explained according to the properties that are favored to be in an ideal lung scaffold, such as in terms of differentiation and engraftment cues, immunogenicity, manufacturability, and long-term storage. These differences are summarised in Table 1 below.

## 5. Potential Manufacturing Methods

A variety of manufacturing methods have been used for tissue engineering of porous structures, such as solvent-casting, foaming, freeze-drying, and particulate-leaching [64]. Particularly in terms of the lung parenchyma, the electrospinning method is widely studied and is deemed effective in producing nanoscale fibers from a solution using electricity [65,66]. In electrospinning, both synthetic and natural polymers can be used to create porous scaffolds comprised of thin nanofibers that are capable of supporting cell attachment, proliferation, and differentiation. However, this method has only been used as an in vitro platform to study the effects of fibrotic lung micro-environments but is yet to be used for bioengineering lung tissues [67].

On the other hand, bioprinting, also known as 3D printing, has recently emerged as a potential source for bioengineering tissues or support structures [68,69,70]. 3D printing can produce lung tissue biomimetics to develop in vitro models and could eventually produce functional tissue for transplantation. However, printing functional synthetic tissues that could recreate the lung structure is still beyond the current capabilities of 3D bioprinting technology [71]. Moreover, currently, there are no published reports of attempts to 3D print lung tissue capable of gas exchange. This could be due to the extremely challenging fabrication of distal lung as the gas exchange barrier is in the order of nanometers, and nozzles used for printing cells are currently in the micrometer range [12].

## 6. Hybrid Materials

Researchers are also working to create hybrid materials to build an optimal lung scaffold whereby the positive attributes of two or more materials are combined to produce a final optimal scaffold that overcomes the limitations of each constituent component. For example, the conducive nature of acellular scaffolds for cell adhesion sites, organizational and differentiation cues, paired with synthetic materials and advanced manufacturing approaches to produce reproducible scaffolds with tunable mechanical properties, together could create a desirable lung scaffold [12]. To create hybrid materials, a good understanding of the anatomy and physiology of the organ is necessary. Park et al. used a hybrid implant consisting of L-lactide and ε-caprolactone, which was synthesized by ring-opening polymerization. An advantage of this matrix is that it is highly porous (80%), which facilitates cells to migrate into the implant. Gelatin coating was also applied to strengthen the matrix, thereby improving cell adhesion on the surface. Moreover, gelatin matrix coated with PCL/TGF-B1 (polycaprolactone/transforming growth factor beta-1) with cultured chondrocytes on the surface showed good results in in vivo experiments, whereby after 8 weeks of implantation, the matrix retained its mechanical properties despite the gradual replacement by native tracheal tissue [72].

## 7. Bioreactor Strategies for Lung Bioengineering

**Figure 2:**
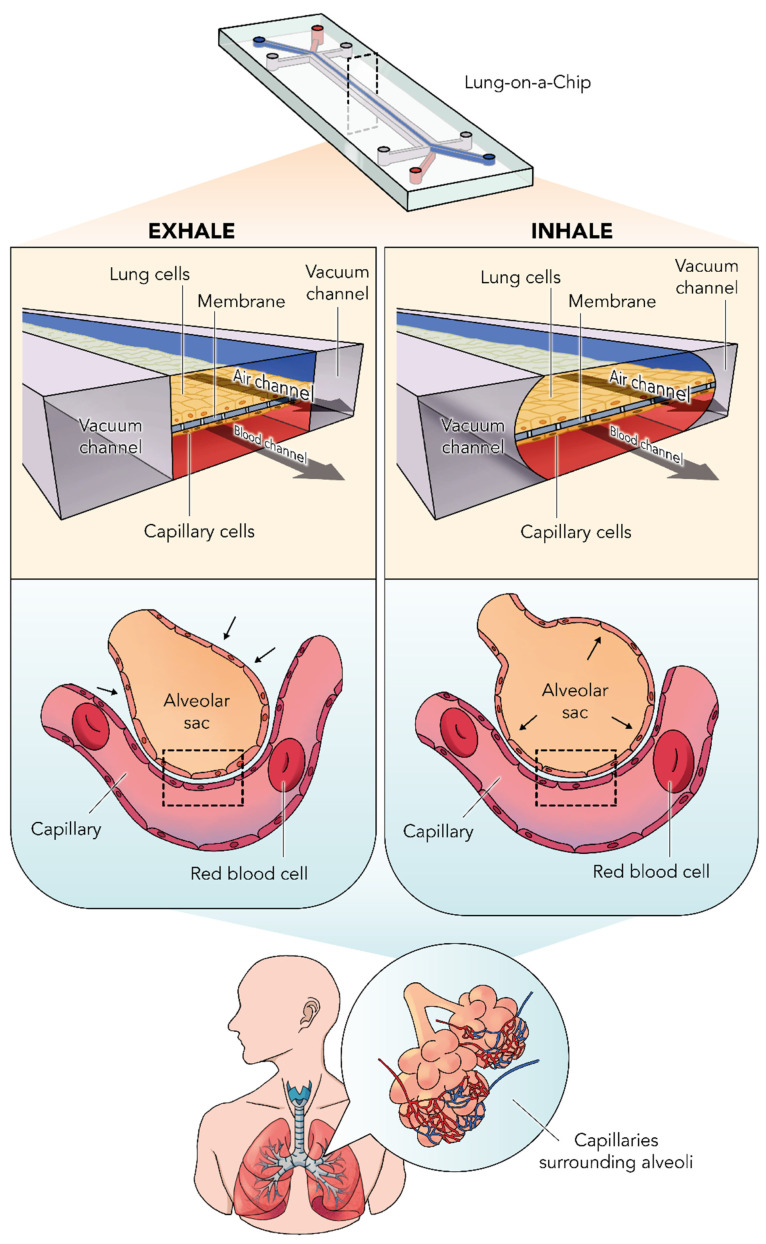
The mechanistic representation of the lung-on-a-chip (emulate chip) model. The principle of biological breathing (inhale–exhale) is reconstructed by applying vacuum to the channels on the sides. During the inhalation phase, this vacuum would cause a stretch on the membrane that represents the alveoli–capillary junction. The dotted line signifies the area where the membrane is stretched. Several drugs were reported to be screened using this technique [80].

Bioreactors in tissue engineering are defined as devices that facilitate biological processes by maintaining physiological parameters at desired levels, improving mass transport rates, and exposing cell-seeded 3D scaffolds to specific physical or biochemical stimuli to support in vitro tissue development or encourage cells to undergo differentiation [73,74]. In terms of lung bioengineering, bioreactors are crucial in cell expansion and differentiation, tissue decellularization and recellularization, engineered tissue culture monitoring, and reconditioning of lungs to make them usable for transplantation [75]. Bioreactors ranging from microfluidic scale to human-sized whole lung systems have been developed recently. Microfluidic, lung mimic, and lung slice cultures have advantages of cost-efficiency and high throughput analyses ideal for pharmaceutical and toxicity studies (Figure 2), whereas perfused rodent whole lung systems can be adapted for mid-throughput studies of lung progenitor cell development, cell behavior, understanding and treating lung injury, and for preliminary work that can be translated to human lung bioengineering [75].

For scalable cell culture and differentiation, bioreactor systems need to provide a minimized gradient in pH, oxygen, nutrients, and adequate surface for anchorage-dependent cells. Rotating bioreactors and microcarrier-based bioreactors have been developed by fulfilling the aforementioned criteria. Ghaedi et al. successfully obtained optimally differentiated iPSCs into type I epithelial cells via rotating bioreactors. This has shown that rotating bioreactors are able to provide a well-maintained polarizing air-liquid interface required to maintain respiratory epithelium differentiation and growth in vitro [76]. On the other hand, since microcarrier-based reactors cater to a large surface-to-volume ratio and allow culture in suspension mode, cellular expansion in a relatively low volume with minimal pH and nutrient gradients is feasible [77].

Bioreactors have also been developed for decellularization and are also amendable by design for recellularization [36,78]. In a bioreactor, decellularized lung scaffold upon cannulation is mounted into the trachea and pulmonary artery; then, the culture medium is perfused at physiological pressure [79]. To recellularize both airway and vascular conduits, a bioreactor must possess independent access lines with integrated pressure transducers to enable flow or volume-based control of pressure. Instead of continuous, pulsatile, perfusion through the vasculature will stimulate heart-driven blood flow; plus, it could confer appropriate nutrient distribution while removing cellular and extracellular waste products. The bioreactor should also provide mechanical ventilation, ideally by negative pressure to avoid lung damage. However, to reverse lung collapse and allow airway recruitment, positive pressure inflation might also be required [75].

Several studies have developed large-scale bioreactors for human size lung tissue engineering, many of which led to the implantation of animal models [81,82]. An organ culture system and protocols to support recellularization of whole acellular human pediatric lung scaffold were developed whereby the bioreactor was used for both decellularization and recellularization. After 30 days of bioreactor culture, type I and type II alveolar epithelial cells and alveolar–capillary junctions were present, and the static compliance of engineered and normal lungs were similar [82]. Furthermore, to culture rhesus macaque lung tissue with bone marrow-derived mesenchymal stem/stromal cells (BM-MSCs) and lung microvascular endothelial cells, a bioreactor providing mechanical stretch and strain by negative pressure ventilation and pulsatile perfusion through the vasculature was utilized. It was found that, after 2 weeks of the culture, the BM-MSCs had grown along the large airways’ lumenal surface, lined the alveolar septae, and resembled simple squamous epithelium [83].

**Figure 3:**
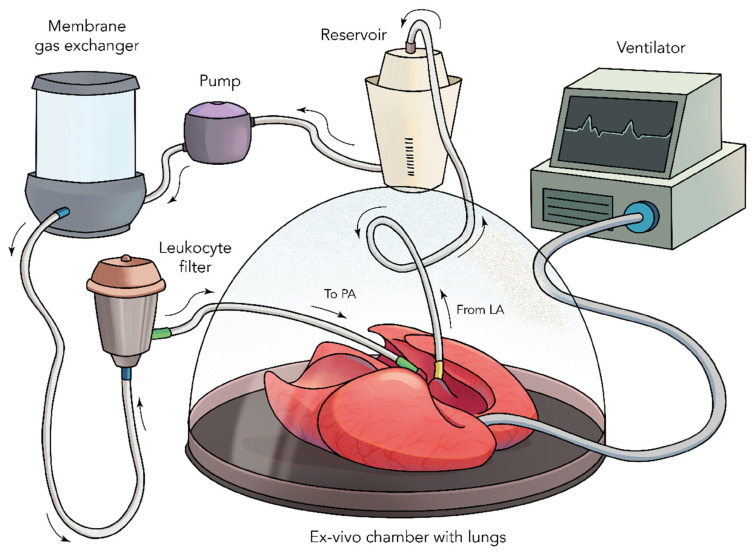
A diagrammatic representation of Ex Vivo Lung Perfused (EVLP) System.

Moreover, ex vivo lung perfused (EVLP) systems have been used in studying lung diseases and as potential therapeutic interventions (Figure 3). In EVLP systems, the lungs are oriented in a horizontal position laying down on a solid surface, whereas, in decellularization/ recellularization chambers, the lungs are held upright and are suspended in medium. Despite the advances made, the maximum amount of time a healthy lung can be maintained ex vivo and transplanted is in the range of 6 h, which has prompted many investigators to avoid using EVLP systems for prolonged lung maintenance or for bioengineering purposes [84]. Although interestingly, a study in a cross-circulation model using native porcine lungs reported an ex vivo period of 36 h, but transplantation was not evaluated as the primary outcome [85]. Since the evolution of lung preservation systems such as Organ Care System from Transmedics™ and XPS™ from XVIVO Perfusion, current clinical trials of lung transplantation to prolong organ preservation and recondition lungs are being undertaken [86,87,88].

## 8. Regulatory and Ethical Implications in the Approach of Lung Bioengineering

Lung tissue bioengineering is a complex process that emphasizes the use of various technologies from scaffold manufacturing to bioreactors to develop functional lung tissue which had been impaired by illness or injury. Likewise, the regulatory and ethical issue in tissue engineering is similarly complex, especially in terms of the translational progression from early benchwork and pre-clinical studies to clinical research.

In the pre-clinical stages, many ethical issues, including responsible reporting, dissemination of results, data integrity, and ensuring that every study conducted is designed to yield results suitable to decide on the next research steps, are required to be considered. It is also important to realize that at any point of research, obtained results might lead not forward, but backward, or in a different direction entirely, which could assist in refining knowledge at an earlier stage or in exploring newly identified possibilities [89]. Furthermore, the use of animal models remains the mainstay in the success of tissue engineering. Although potential alternatives such as computer modeling and body-on-a-chip organoid arrays are being further studied, these approaches still have limitations and require considerable further development. Therefore, researchers should adhere to the principle of modest translational distance, which refers to the number and size of inferential leaps from animals to humans to remain minimal in selecting animal models for in vivo studies [90]. Moreover, during the recellularization process, the use of embryonic stem cells also provokes major ethical controversies. To avoid being tangled in this issue, researchers opt to use iPSC that are derived from reprogramming somatic cells to a stem-like state.

Although significant information through in vitro and in vivo studies could be obtained, it would not be sufficient to fully elucidate the physiological and biochemical interactions that occur within a human being. Hence, prior to clinical trials, a clinically relevant large animal model that examines short- and long-term outcomes are crucial to be conducted [91]. Regulatory frameworks and good manufacturing practices (GMP) standards establishment is crucial to prioritizing patient safety [92,93]. To date, the evaluation criteria used for bioengineered lung tissue prior to clinical trials remain unknown, but the use of parameters comparable to those used in EVLP may be a good first indication. It is critical for academic researchers, clinicians, regulatory bodies, and industries to work with one another in establishing these new frameworks [12].

To produce transplantable lungs using recellularization approaches, it is important to select a donor source for generating decellularized scaffolds in which human organs are favorable candidates due to the issue of immunogenicity. However, most studies that use human lungs for decellularization do not meet the clinical criteria for transplantation. In addition, structural lung diseases such as pulmonary fibrosis or emphysema are often an issue in donor organs [94]. In these instances, the lungs donated after cardiac death (DCD) approach can be further explored. DCD lungs are an underutilized resource for a dwindling donor lung transplant pool [95]. Currently, DCD lungs are assessed using ELVP before transplantation, but most do not reach the minimum requirement, such as low blood gas values [96]. Hence, if the ethical committee of a particular country allows the use of DCD lungs, bioengineering strategies can be applied to generate reproducible functional lung tissue.

## 9. Conclusions

The advancement in technology has been a propellor in the field of complex tissue engineering, allowing the production of new treatment prospectus in terms of healthcare. Tissue engineering is a multivariate field that requires collaboration between engineering, biology, medicine, chemistry, and emerging novel multidisciplinary technologies. One of the greatest barriers to the generation of an ideal bioengineered lung is surely the complexity and unique architecture of the organ itself. Without knowing and understanding the precise control and direct angiogenesis of creating intricate pulmonary vasculature, development in lung tissue engineering could not be able to move forward. Hence, more investigations are being conducted using human-sized bioreactors for lung decellularization and recellularization along with EVLP systems. The goal is to produce a bioartificial lung as an alternative to traditional donor lungs in patients suffering from end-stage lung disease and thus meeting the demand of patients on the waiting list. More research is needed in translating pre-clinical studies to clinical studies in the hopes of presenting the implantation of the bioartificial lung as a functional treatment option in the near future.

## Figures and Tables

**Table 1:** Differences between properties of acellular and artificial scaffolds.

Acellular Scaffold	Properties	Artificial Scaffold
Native integrin-binding site is retained	Differentiation and engraftment cues	Specific integrin-binding site is absent. Must be engineered into scaffolds.
Removal of antigen during decellularization	Immunogenicity	Varies depending on material used
Native architecture largely retained	Manufacturability	Complex architecture possible
Large variability between donor scaffolds	Similarity with donor	Precise control possible
Degradation over long term storage	Long term storage	Improved storage stability
